# Bibliometric and LDA analysis of the correlation between childhood obesity and precocious puberty (2005–2025)

**DOI:** 10.3389/fped.2026.1846658

**Published:** 2026-07-27

**Authors:** Kang Li, Yingying Xiang, Li Zhang, Li Shi, Wei Qian, Mingzhe Zhu

**Affiliations:** 1 School of Public Health, Shanghai University of Traditional Chinese Medicine, Shanghai, China; 2 Institute of Digestive Diseases, Longhua Hospital, Shanghai University of Traditional Chinese Medicine, Shanghai, China

**Keywords:** bibliometric analysis, childhood obesity, citespace, latent dirichlet allocation, precocious puberty

## Abstract

**Background:**

Growing evidence supports a close association between childhood obesity and precocious puberty (PP). However, there remains a lack of bibliometric research specifically examining the factors and trends within this intersecting field. A systematic bibliometric analysis would provide a comprehensive understanding of the current research landscape, identify key contributors and emerging trends.

**Methods:**

On June 2, 2026, relevant studies published between 2005 and 2025 were retrieved from the Web of Science Core Collection (WoSCC), Scopus and PubMed databases. Using the bibliometrix R 4.5.1, CiteSpace 6.4.R1, and VOSviewer 1.6.20, we conducted a visual analysis of dimensions such as publication volume, countries, institutions, authors, journals, and keywords. We employed the Latent Dirichlet Allocation (LDA) method to reveal the underlying thematic structure and track its temporal evolution.

**Results:**

A total of 2506 publications were included, involving 9,843 authors and spanning 664 academic journals. The annual publication volume increased steadily, with the United States and China as the major contributing countries. Harvard University led institutional output, followed by the University of California System and Leipzig University. Chen Y was the most prolific author with 42 publications. *Journal of Pediatric Endocrinology & Metabolism* was the most productive journal in this field. “Obesity” (1,179) and “puberty” (1,031) were identified as the core research keywords. LDA analysis yielded 15 topics, which were grouped into four clusters. These clusters respectively cover adrenal endocrine function and pubertal physiology, obesity-related metabolic complications and multisystem damage in children, the link between childhood obesity and pubertal timing, and relevant upstream etiological factors.

**Conclusion:**

This study applies bibliometric analysis and LDA topic modeling to trace the development of research on the association between childhood obesity and PP over the past two decades. It systematically reviews research progress in this field, clarifies publication trends, the global research landscape, and patterns of thematic evolution, thereby providing an important academic reference for clinicians and scholars in the field of pediatrics to identify research hotspots and cutting-edge directions.

## 1 Introduction

Childhood obesity has become an increasingly serious global public health challenge. It is not merely a matter of being overweight, but a chronic disease characterised by the abnormal accumulation of adipose tissue (1, 2). The World Health Organization (WHO) defines it as “abnormal or excessive fat accumulation that may impair health” (2). According to a WHO report, the global obesity rate among children and adolescents aged 5–19 years has increased from 2% to 8% since 1990 until 2022 (3). Childhood obesity may persist into adulthood and is closely associated with increased risks of chronic noncommunicable diseases like cardiovascular disease, type 2 diabetes, cancer, as well as premature death (3, 4). It also triggers psychological issues such as low self-esteem and anxiety, placing a heavy long-term burden on individual health and healthcare systems.

PP refers to the premature onset of puberty, characterized by early development of secondary sexual characteristics and physical maturation. As a pediatric endocrine disorder, it can be classified based on whether the hypothalamic-pituitary-gonadal (HPG) axis is activated, distinguishing central precocious puberty (CPP) from peripheral precocious puberty (PPP). Puberty onset before age 8 in girls and before age 9 in boys is typically defined as PP (5). Notably, the incidence of PP exhibits significant gender and regional disparities (6–8), with girls showing a much higher prevalence than boys. Furthermore, the detection rate of childhood PP is relatively higher in areas with higher economic development and rapid urbanization. Alongside the rising prevalence of childhood obesity, the global incidence of childhood PP has also shown a marked increase, currently ranging from 0.1‰ to 0.2‰ and continuing to rise annually (9). PP accelerates skeletal maturation and impairs final adult height; it can also lead to psychological problems such as low self-esteem, and increase the risk of developing chronic conditions in adulthood, including metabolic syndrome, cardiovascular disease, polycystic ovary syndrome and hormone-related cancers (10, 11).

Epidemiological data indicate that childhood obesity and PP are highly co-occurring conditions and share similar health risks, and obesity is a major independent risk factor for PP (12–15). Similarly, PP can also act as an independent risk factor for childhood obesity (16), suggesting a bidirectional association between the two. The combined effects of obesity and PP amplify lifelong health risks for children (9, 11, 17). This suggests a potential association between the two; existing research has explored this association from multiple perspectives, including endocrine regulation, molecular genetics, environmental exposure, and individual psychological and social factors. However, research is scattered across multiple disciplines, including paediatrics, endocrinology, epidemiology, nutrition and psychology, making it difficult for individual researchers to keep abreast of all developments comprehensively. At present, numerous bibliometric studies at home and abroad have systematically analyzed the research status, hotspots and evolutionary trends of childhood obesity alone, forming a well-established research system for this single disease (18, 19). Nevertheless, there is still a lack of systematic bibliometric research focusing specifically on the comorbidity of childhood obesity and precocious puberty. The overall knowledge framework, research hotspots and future development trends in this field remain unclear. Accordingly, it is essential to conduct comprehensive bibliometric and thematic analysis to fill this research gap.

Bibliometrics is a field that employs mathematical and statistical techniques to conduct quantitative and qualitative analyses of scholarly literature, enabling the elucidation of patterns of disciplinary evolution and distribution-citation dynamics within scientific literature (20–22). LDA is a probabilistic generative model widely used in scientometrics and the social sciences, capable of objectively identifying latent thematic structures within large-scale text corpora (23). By combining bibliometric analysis with LDA topic modeling, we analyzed global literature on the association between childhood obesity and PP. This approach will help researchers grasp the current research status, identify hot topics, predict future trends, and facilitate further in-depth investigations.

## 2 Data sources and research methods

### 2.1 Data sources

This study retrieved data from internationally recognized high-quality academic literature index platforms: WoSCC, Scopus, and PubMed databases. The search was updated as of January 31, 2026. A search strategy was developed based on Medical Subject Headings (MeSH) and relevant literature characteristics. The specific search formulas for each database are as follows: In WoSCC: “TS = (child* OR pediatric* OR adolescen* OR juvenil*) AND TS = (overweight OR obes* OR adiposit* OR “BMI” OR “adipose tissue”) AND TS = (“precocious puberty” OR “early puberty” OR puberty* OR “central precocious puberty” OR CPP OR sexual precoci* OR sexual matur*)”; in Scopus: “TITLE-ABS-KEY ((child* OR pediatric* OR adolescen* OR juvenil*) AND (overweight OR obes* OR adiposit* OR BMI OR “adipose tissue”) AND (“precocious puberty” OR “early puberty” OR puberty* OR “central precocious puberty” OR CPP OR sexual precocit* OR sexual matur*))”; PubMed search: “(child* OR pediatric* OR adolescent* OR juvenile*) AND (overweight OR obese* OR adiposity* OR BMI OR “adipose tissue”) AND (“precocious puberty” OR “early puberty” OR puberty* OR “central precocious puberty” OR CPP OR sexual precocit* OR sexual matur*)”.

**Figure 1:**
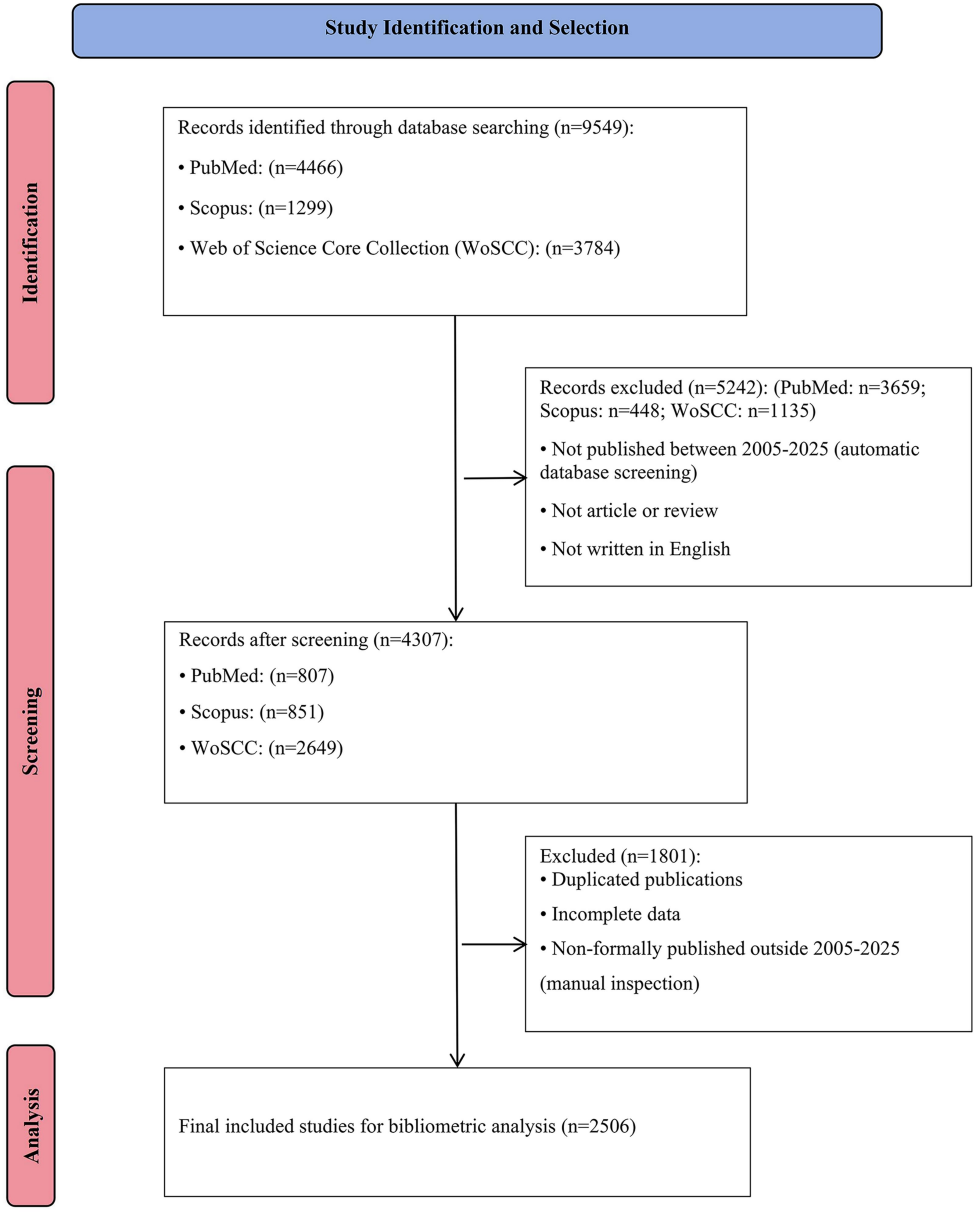
Article screening flowchart. Some online pre-released articles without formal publication were manually excluded upon full-text inspection.

Search parameters were standardized as follows: language “English”, time span 2005–2025, and document types limited to “Article” and “Review”. All retrieved records were exported in Plain Text File or BibTeX formats, converted into RIS format, and merged using the bibliometrix package in R software. Duplicates were first automatically detected and removed according to DOI, title, authors and publication year. Afterwards, we manually rechecked all remaining entries to eliminate residual duplicates and publications outside the 2005–2025 time frame. During the literature screening process, data extraction was performed independently by two researchers. Any uncertainties or discrepancies were resolved by consensus with a third reviewer, and necessary adjustments were made. Ultimately, 2,506 valid documents were included as the research sample for subsequent bibliometric and thematic analysis. The specific search process is illustrated in Figure 1.

### 2.2 Research methods

This study employs CiteSpace 6.4.R1, VOSviewer 1.6.20, the R 4.5.1 programming language, and the LDA topic model to conduct multidimensional visualization analysis and in-depth mining of research data. Their synergistic application provides critical technical support for systematic dataset analysis and precise extraction of research patterns.

CiteSpace is a professional bibliometric and knowledge graph visualization tool capable of mining vast amounts of scientific literature to identify research hotspots, knowledge evolution and key nodes, while intuitively presenting collaboration networks and keyword emergence trends, thereby providing a basis for understanding the landscape of a discipline (21).

VOSviewer is a tool for constructing and visualising bibliometric networks. It uses cluster analysis to clearly illustrate collaborative relationships between keywords, authors and institutions, thereby enabling the efficient identification of research hotspots and thematic characteristics (24).

The Bibliometrix plugin for R is open-source software that enables structured analysis of massive literature datasets. It facilitates identifying publishing trends, delineating disciplinary boundaries, and discovering key scholars and institutions (21, 25).

LDA is an unsupervised probabilistic generative model widely applied in scientometrics, information science, and social sciences. It automatically extracts latent themes from large-scale text corpora, effectively revealing the semantic structures and knowledge logic underlying literature data (23, 26). This study employed a full-grid search method to conduct a full-combinatorial evaluation of the number of candidate topics [K = (5, 10, 15, 20, 25)] and the hyperparameters α (0.01, 0.05, 0.1) and β (0.005, 0.01, 0.05, 0.1), yielding a total of 60 sets of parameter results. Two core evaluation metrics, perplexity and UMass topic coherence, were used for model selection. Lower perplexity indicates better fitting performance of the model on the corpus; UMass coherence was calculated based on the top 10 high-frequency words of each topic, and values closer to 0 represent higher semantic coherence of topics. This study prioritized topic coherence as the primary criterion and took perplexity as the auxiliary reference. The detailed results of all parameter combinations are summarized in Supplementary Table S1 and visualized in Supplementary Figure S1. As shown in Supplementary Figure S1, the perplexity continuously decreased with the increase of K, with a remarkable decline from K = 5 to K = 15 and a markedly slowed decreasing rate when K > 15. Meanwhile, the UMass topic coherence reached the optimal value at K = 15, and the corresponding perplexity remained at a relatively low level. Further increasing the number of topics could not substantially improve topic coherence, and would also lead to overly fragmented topics and poor interpretability. After comprehensive evaluation, K = 15 was finally determined as the optimal number of topics. With the optimal number of topics fixed at K = 15, this study further conducted refined hyperparameter optimization and Bootstrap resampling stability tests, ultimately determining the optimal priors as *α* = 0.05 and *β* = 0.01. All subsequent topic mining and quantitative analyses were conducted based on this set of parameters.

## 3 Results

### 3.1 Dataset overview, publication volume and citation trends

#### 3.1.1 Dataset overview

**Figure 2:**
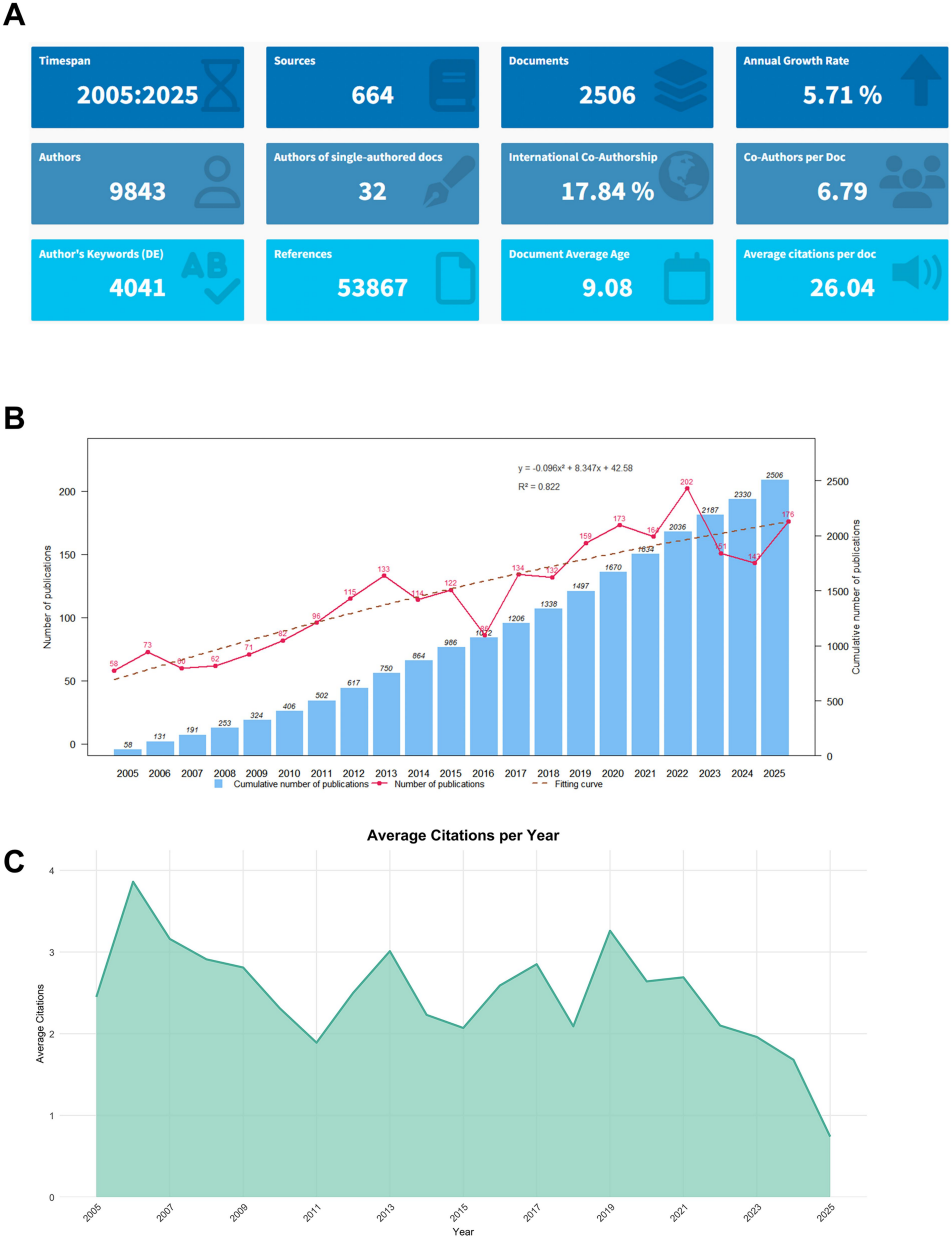
Basic information, publication volume and citation trends. **(A)** Basic information of research on childhood obesity and PP. **(B)** Annual number of publications—showing the growth trend. **(C)** Average citations per year—showing the impact trend. PP-Precocious Puberty.

Using the R-bibliometrix bibliometric tool for analysis, the research profile from 2005 to 2025 was as follows (Figure 2A): The analysis included a total of 2,506 publications, sourced from 664 distinct journals and books, indicating a broad publishing foundation within this research field. Academic output maintained a stable annual growth rate of 5.71%, with an average publication lifespan of 9.08 years, reflecting solid historical accumulation and sustained scholarly expansion. Each paper received an average of 26.04 citations, and the entire literature corpus garnered 53,867 total citations, fully demonstrating this field's high influence.

Author analysis revealed a highly collaborative culture within this research domain. A total of 9,843 authors contributed to the research, with an average of 6.79 co-authors per paper. Single-author publications were relatively rare, with only 32 authors producing solo works. International research collaboration was particularly prominent, accounting for 17.84% of all co-author relationships. Furthermore, the field exhibited 4,041 author-defined keywords, reflecting the high dispersion of research topics and the diversity of research perspectives.

#### 3.1.2 Publication trend

The temporal variation in publication volume effectively reflects research trends and progress within a field. An analysis of annual publication trends from 2005 to 2025 was presented in Figure 2B. According to the quadratic fitting curve, despite minor fluctuations, the rising trend in annual publication volume remained unchanged (y = −0.096×^2^ + 8.347x + 42.58, R² = 0.822). During the initial phase (2005–2009), annual publication numbers fluctuated at a low level between 58 and 73. From 2010 onwards, publications entered a phase of sustained growth, rising year on year amidst slight fluctuations, reaching a peak of 202 in 2022. Following 2022, annual output declined slightly before rebounding to 176 papers in 2025, with the cumulative total for the entire period steadily increasing to 2,506 papers. In summary, this research field gradually evolved from an emerging, nascent discipline into a mature academic field. Although annual output experienced short-term fluctuations in recent years, the long-term trend of cumulative literature growth remained robust and confirmed the continuous rise in its global academic influence.

#### 3.1.3 Citation trends

Citation trends reflect annual citation fluctuations of publications and research fields, and they quantify the evolving popularity and long-term academic influence of relevant topics.

Figure 2C illustrated the average annual citation frequency of papers published in this field between 2005 and 2025. There were significant temporal variations in the average annual citation impact per paper, with an overall downward trend. The average annual citations per paper peaked in 2006, reaching the highest level recorded during the entire study period. Since then, the annual average citation rate generally followed a downward trend, though several periods of slight recovery occurred and formed minor peaks in 2013, 2017 and 2019; following 2019, the annual average citation rate continued to decline and reached its lowest point for the entire period in 2025. This trend was influenced by the citation ageing effect: recently published literature, due to its shorter citation window, often receives fewer citations; this does not necessarily indicate a decline in academic influence. The trend also suggests that the citation volume of earlier publications gradually decreases over time; therefore, when conducting longitudinal comparisons of citation metrics, one must fully account for the differences arising from the year of publication.

### 3.2 National and institutional publication output and collaboration

#### 3.2.1 National publication output and collaboration trends

National publication analysis revealed the global landscape of research linking childhood obesity to PP. A total of 76 countries and regions contributed relevant publications. The top 10 countries/regions ranked by the publication count of corresponding authors are presented in Table 1. All statistics in Table 1 adopt the corresponding author-based counting method, whereby each publication is exclusively attributed to the country of the corresponding author.

**Table 1:** Top 10 countries/regions by number of publications.

Rank	Countries/Regions	Articles	Articles %	SCP	MCP	MCP %
1	USA	516	20.6	429	87	16.9
2	China	260	10.4	218	42	16.2
3	Brazil	208	8.3	183	25	12
4	Italy	127	5.1	111	16	12.6
5	Spain	113	4.5	80	33	29.2
6	United Kingdom	108	4.3	75	33	30.6
7	Germany	100	4	77	23	23
8	Korea	73	2.9	67	6	8.2
9	Turkey	67	2.7	67	0	0
10	Denmark	62	2.5	41	21	33.9

SCP, single country publications; MCP, multiple country publications.

In terms of publication volume, the United States was the primary contributor, having published 516 papers, accounting for 20.6% of the total. Among these, 429 were single-country publications (SCP) and 87 were multi-country collaborative publications (MCP), with MCPs constituting 16.9% of its national output. China followed closely with 260 publications (10.4%), where MCPs constituted 16.2%. Brazil, Italy, and Spain contributed 208, 127, and 113 publications respectively, forming the core global research forces. Significant variations existed in the proportion of domestic versus international collaboration across countries. Some countries were highly dependent on international cooperation; Denmark, the United Kingdom and Spain featured the highest cross-border collaboration ratios, with MCP percentages reaching 33.9%, 30.6% and 29.2% respectively. By contrast, Turkey had zero international collaborative articles (MCP = 0, MCP% = 0), relying on domestic independent research.

**Figure 3:**
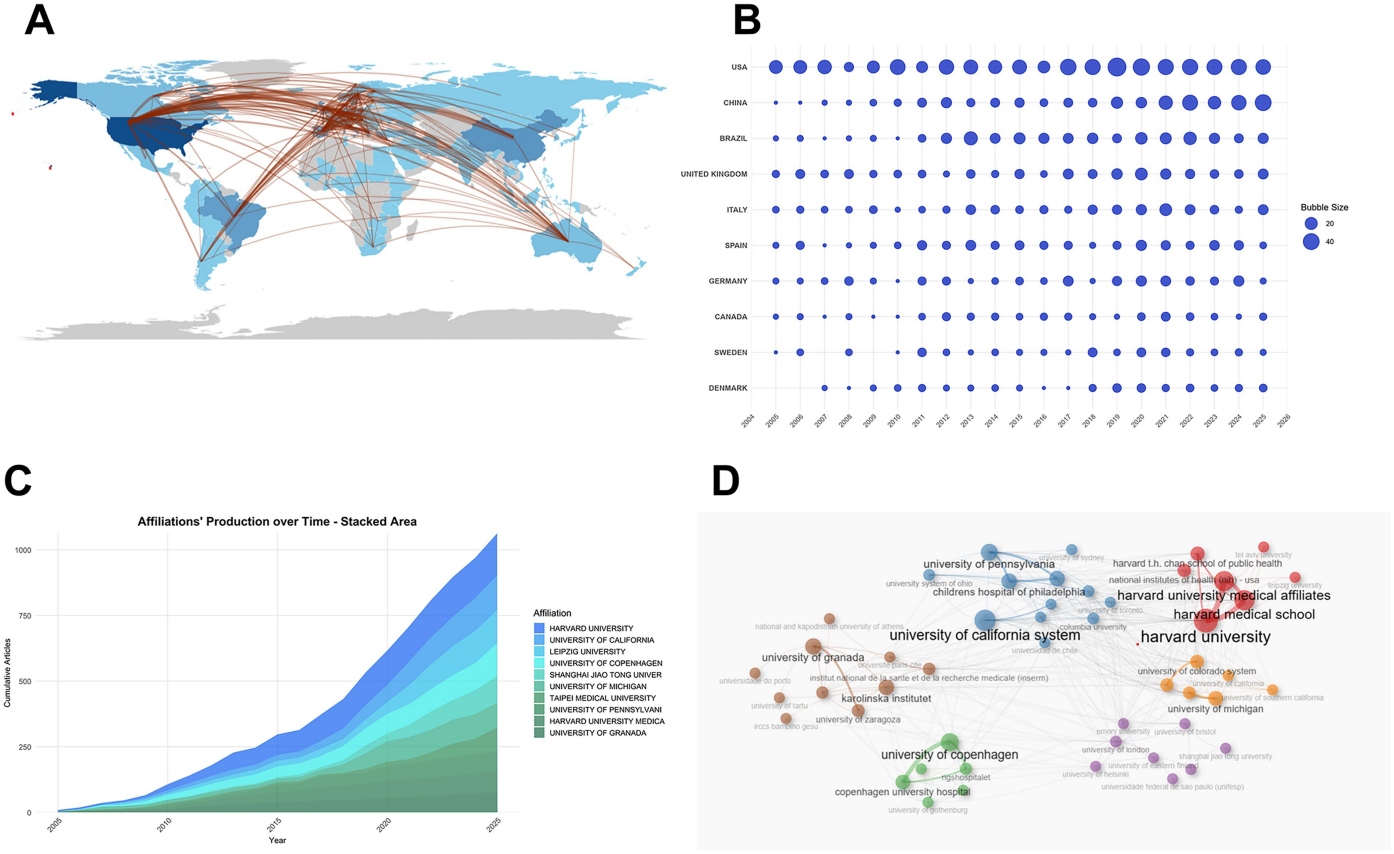
National and institutional publication output and collaboration. **(A)** World map of country collaboration. **(B)** Bubble plot of annual publication output for the top 10 countries. **(C)** Temporal trends in publications by the top 10 institutions **(D)** Institutional collaboration network. Node size represents publication volume, line thickness indicates collaboration intensity, and color denotes clustering groups.

The visualization of inter-country collaboration networks (Figure 3A) provided a clearer picture: the global cooperation network formed dense transnational connections centered around the United States, China, and core European nations. Cooperation ties clustered densely across European countries to form tight intracontinental partnerships, while frequent cross-continental cooperation was observed not only between the United States and Brazil but also between the US, China and Australia.

Publication rankings by corresponding authors and cross-country collaboration networks clarified national research strengths and global cooperation patterns, yet failed to capture overall national publication volume and its yearly dynamic shifts. Accordingly, total publications were counted using the full counting method based on all-author affiliations, the top 10 productive countries were identified, and a bubble chart was generated to illustrate the annual evolution of national output (Figure 3B), where bubble size represented annual publication volumes. During the entire study period, the United States kept its long-term leading position in total output; its publications expanded rapidly from 2018 to 2020 and remained at the highest level afterwards. Major European nations including the United Kingdom, Italy, Spain, Germany, Sweden and Denmark maintained steady overall publication performance across the two decades with mild annual volatility. China started with minimal publications in the early stage, yet its output climbed remarkably after 2018 and saw continuous expansion in subsequent years. Brazil presented prominent output expansion around 2013 and kept an overall upward trend thereafter. Although Canada showed small periodic fluctuations, all ten featured countries witnessed gradual publication growth across the research period, which reflected steadily rising global research activity focusing on childhood obesity and precocious puberty.

#### 3.2.2 Institutional publication output and collaboration trends

A total of 2,781 institutions participated in research within this field. The top 10 institutions by publication volume, as shown in Table 2, clearly revealed the core research institutions in this domain. All institutional publication counts in this study were calculated using the full counting method, in which all collaborating institutions were counted separately for each multi-institutional paper. Publication output exhibited significant concentration, with core research capabilities primarily concentrated in developed countries in Europe and America. Harvard University led with the highest publication count, having published 160 papers. The University of California System and Leipzig University followed closely, publishing 132 and 126 papers respectively.

**Table 2:** Top 10 institutions by number of publications.

Rank	Institution	Publications	Country
1	Harvard University	160	USA
2	University of California System	132	USA
3	Leipzig University	126	Germany
4	University of Copenhagen	125	Denmark
5	Shanghai Jiao Tong University	102	China
6	University of Michigan	94	USA
7	Taipei Medical University	91	China
8	University of Pennsylvania	80	USA
9	Harvard University Medical Affiliates	77	USA
10	University of Granada	75	Spain

Cumulative publication output refers to the total accumulated number of published papers over successive years and reveals the overall growth tendency of research productivity in a field. Figure 3C illustrated the cumulative publication output of the top ten institutions in this field, clearly showing the temporal evolution of research output between 2005 and 2025. The results indicated that Harvard University consistently led in terms of cumulative publication output and established itself as the most pivotal research entity in this field; the University of California system and the University of Pennsylvania followed closely behind and formed a stable pillar of research in North America. The number of publications from each institution grew significantly between 2009 and 2014, with growth accelerating after 2015, which led to a substantial overall increase in cumulative publications by 2025. Meanwhile, institutions in the Asia-Pacific region, represented by Shanghai Jiao Tong University and Taipei Medical University, as well as three European institutions including Leipzig University, University of Copenhagen and University of Granada, continued to produce research outputs and jointly drove the continuous enhancement of research vitality in this field.

Figure 3D presented a visual analysis of the institutional collaboration network. The results showed that North American institutions, represented by Harvard University, occupied a core position. They led in both publication output and collaboration intensity, constituting a dominant global research force. Major European universities, such as the University of Leipzig and the University of Copenhagen, formed several robust collaborative communities, demonstrating distinct regional research characteristics. Institutions in the Asia-Pacific region, represented by Shanghai Jiao Tong University in China, rapidly enhanced their research capabilities and became an important component of regional collaboration; however, direct collaboration with the core clusters in Europe and the United States remained relatively limited. The overall collaboration network exhibited close internal connections within clusters and relatively sparse links between clusters, reflecting both the long-standing leading position of developed nations in this field and the fact that the global research landscape is evolving toward greater diversity and inclusivity.

### 3.3 Author and journal publication characteristics

#### 3.3.1 Author publication and collaboration trends

A total of 9,843 authors participated in research within this field. Table 3 listed the top 10 prolific authors (including ties) worldwide ranked by total publications (TP). Chen Y from China led with 42 publications (TP = 42; H-index = 13). Pereira A from Brazil occupied second place (TP = 35, H-index = 13), followed by Moreno L from the USA in third place (TP = 34, H-index = 21), whose H-index of 21 and total citations (TC = 1,227) were the highest among all listed authors. Kiess W from Germany and Wang X from China ranked fourth and fifth with TP of 33 and 29 respectively. Notably, Ortega F from Spain published only 23 papers and ranked eighth based on publication quantity, but his total citations reached as high as 1,111. Together with Moreno L, they obtained remarkably high citation counts, demonstrating outstanding academic influence.

**Table 3:** The most productive ten authors (including tied ranks).

Rank	Author	TP	Country	H-index	G-index	TC	PY start
1	Chen Y	42	China	13	25	707	2017
2	Pereira A	35	BRAZIL	13	26	714	2014
3	Moreno L	34	USA	21	34	1,227	2006
4	Kiess W	33	Germany	19	27	783	2008
5	Wang X	29	China	14	23	536	2007
6	Mericq V	25	Chile	13	25	632	2008
7	Vogel M	24	Germany	14	21	461	2017
8	Ortega F	23	Spain	18	23	1,111	2006
9	Jürimäe J	22	Estonia	12	21	456	2006
10	Kim J	21	South Korea	12	21	476	2005
11	Peterson K	21	USA	16	21	743	2005
12	Wang Y	21	China	11	21	623	2012
13	Zhang Y	21	China	9	19	371	2015

TP, total publications; TC, total citations; PY start, publication year start.

The H-index evaluates long-term academic level; higher values indicate stronger comprehensive research capability. The G-index measures high-impact landmark achievements; higher values mean more high-quality papers.

**Figure 4:**
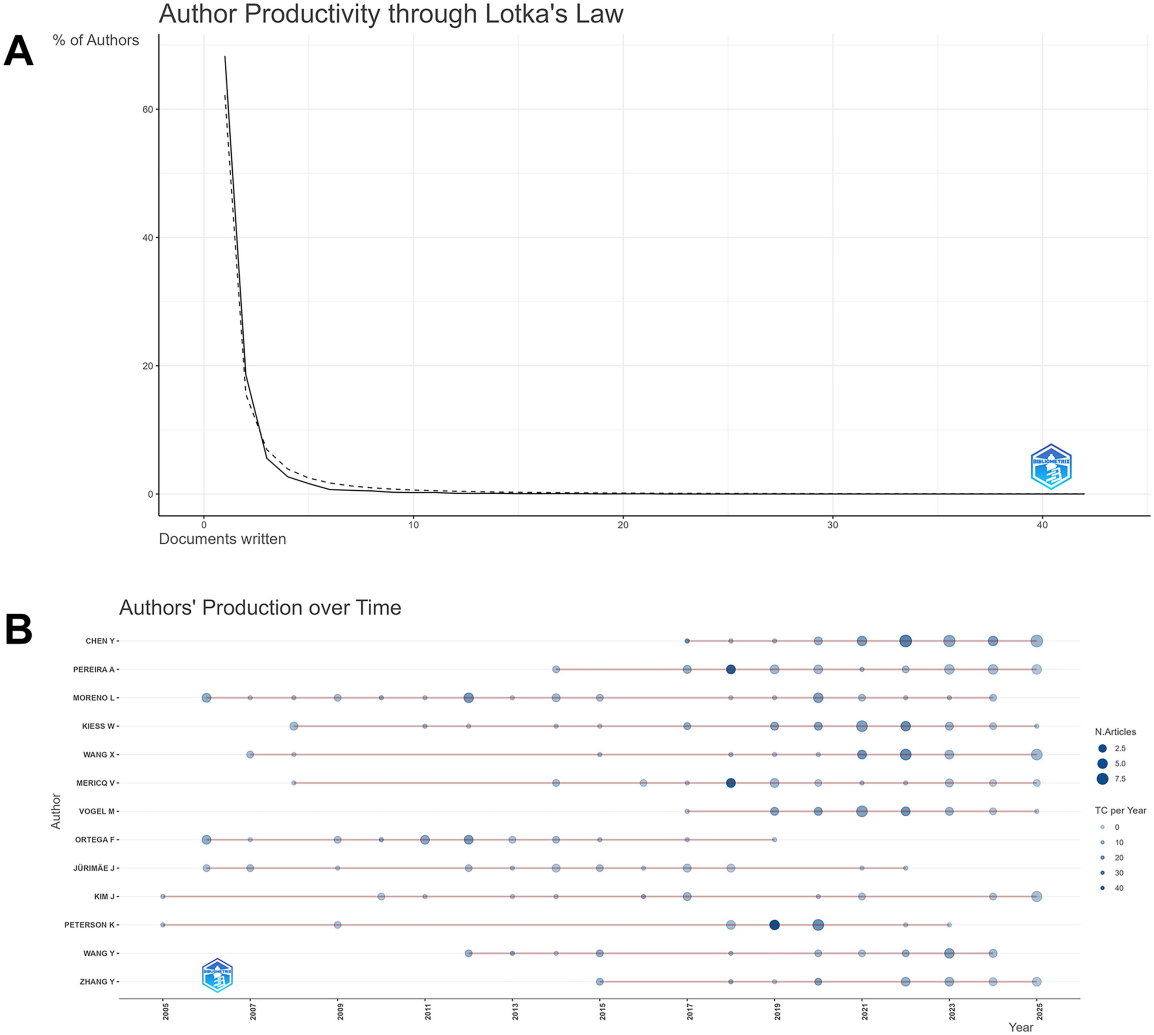
Author publication characteristics. **(A)** Lotka's law. The horizontal axis represents the number of publications per author, while the vertical axis shows the proportion of authors corresponding to each publication volume. The solid line depicts the actual distribution of author output within this research field, whereas the dashed line indicates the theoretical distribution predicted by Lotka's Law (also known as the inverse square law of scientific productivity). **(B)** Authors' production over time chart. Blue data points represent authors' annual publication records, with point size corresponding to the number of articles published (N. Articles) and color intensity reflecting the average citation frequency per year (TC per Year) for that year's publications. Darker blue indicates higher academic impact for papers published in that year.

Lotka's Law can quantify author productivity and classified core research groups. Figure 4A illustrated the distribution characteristics of author productivity in research on childhood obesity and precocious puberty based on Lotka's Law. The calculated β was 2.68, exceeding the classical reference value of 2.00; this finding indicated a higher proportion of low-output authors and a smaller share of prolific contributors alongside weak aggregation of core researchers. As observed from the curves in Figure 4A, authors with publications close to zero accounted for more than 60% of all researchers. The proportion of authors declined rapidly as individual publication counts rose from zero to the maximum horizontal-axis value of 40, and the share of authors approached zero near the 40-publication mark. The overall distribution revealed that a small number of high-yield authors did not dominate total publications in this field. In summary, this research field had extensive participation but no prominent core author group, suggesting that it was still in the developmental stage.

After setting the minimum number of publications to 21, we identified 13 active high-productivity authors within the top 10 rankings including ties. Figure 4B showed the temporal distribution of annual publication volume and academic influence among these core authors. As top-ranked senior scholars in this field, Moreno L and Ortega F published their first papers in 2006. Their publication history spanned over 15 years with steady outputs, acting as long-term academic leaders of the field. Chen Y and Wang X published their first works from 2016 to 2017 and continued publishing until around 2025; some of their articles obtained high annual citations reflected by larger citation dots and they gradually became core contributors in the later development of the field. Pereira A and Merico V achieved remarkable annual outputs, represented by larger publication nodes in several years; yet their publications were intermittent with obvious gaps.

#### 3.3.2 Trends and thematic changes in journal publications

These articles were published in 664 journals. Among the top ten journals by publication volume (Table 4), *Journal of Pediatric Endocrinology & Metabolism* led with 99 articles, followed closely by *Frontiers in Endocrinology* (78 articles) and *Hormone Research in Paediatrics* (70), further highlighting the field's robust research foundation in endocrinology, nutrition, and pediatric endocrinology. It was worth noting that, despite their relatively low output, the *International Journal of Obesity* (38) and *Pediatric Obesity* (37) had total citation counts of 3,112 and 1,610 respectively, indicating higher academic quality and influence. This indicated that the academic quality of papers published in these journals was superior, granting them greater scholarly influence within the field. Based on the 2025 journal impact factors (JIF), *Journal of Clinical Endocrinology & Metabolism* (5.2), *Pediatric Diabetes* (5.2) and *Nutrients* (5.1) ranked highest. Overall, studies in this field were widely published in both specialized journals and high-impact mainstream journals, demonstrating a focused, high-quality publication pattern.

**Table 4:** The top 10 journals with the most publications.

Rank	Sources	Articles	IF (2025)	H-index	G-index	TC	PY start
1	Journal of Pediatric Endocrinology & Metabolism	99	1.2	20	29	1,274	2009
2	Frontiers in Endocrinology	78	4.6	20	30	1,105	2012
3	Hormone Research in Paediatrics	70	2.9	22	34	1,341	2010
4	Journal of Clinical Endocrinology & Metabolism	48	5.2	16	21	573	2020
5	BMC Pediatrics	45	2.1	17	27	847	2013
6	Nutrients	41	5.1	12	15	334	2016
7	International Journal of Obesity	38	3.9	29	41	3,112	2005
8	Pediatric Obesity	37	2.8	17	35	1,610	2012
9	Pediatric Diabetes	33	5.2	17	25	718	2005
10	Journal of Endocrinological Investigation	31	3.9	15	21	511	2005

IF, impact factor (JCR 2025); TC, total citations; PY start, publication year start.

**Figure 5:**
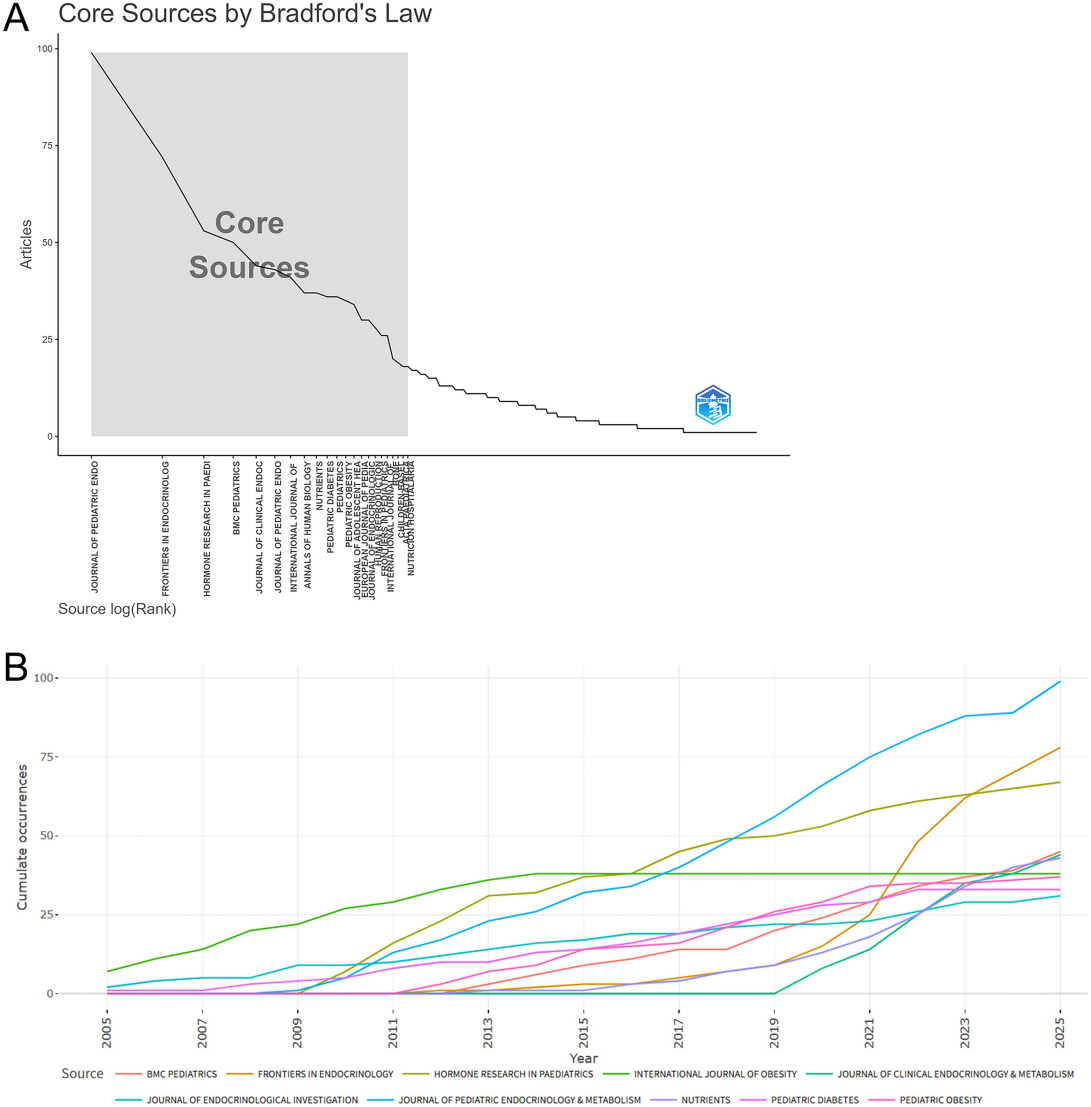
Journal publication characteristics. **(A)** Bradford's law. The horizontal axis shows journals ranked by the number of publications, while the vertical axis shows the number of papers for each journal. The curve exhibits the typical characteristics of a Bradford distribution: a small number of core journals (the shaded gray area) account for a disproportionately high share of the total number of papers, while the publication productivity of journals in the middle and outer zones decreases progressively, confirming the high concentration of research output in this field. **(B)** Cumulative occurrences of journals over time of the top 10 journals (including ties) charted. The horizontal axis represents publication years (2005–2025), while the vertical axis shows each journal's cumulative publication frequency (cumulative occurrences). Different colored lines correspond to distinct core journals. Abbreviation: TC-Total citations.

Bradford's Law describes the concentration-dispersion distribution of disciplinary literature in journals, which helps screen core journals and quantitatively assess the aggregation of published literature. According to Bradford's Law, the distribution of scientific journal articles followed a specific pattern, as clearly illustrated in Figure 5A. The horizontal axis represents the logarithmic rank of journals sorted in descending order by publication volume, while the vertical axis denotes the total number of articles published by each corresponding journal. The curve exhibited a classic “steep decline followed by a gradual plateau” trend. The gray-shaded “Core Sources” region on the left defined the core group of journals in this field. A small number of journals, such as *Journal of Pediatric Endocrinology & Metabolism, Frontiers in Endocrinology*, and *Hormone Research in Paediatrics*, significantly led in publication volume and published most of the core research findings within the field. As the journal ranking progressed, publication volume rapidly decreased and gradually stabilized. This distribution pattern fully aligned with Bradford's Law, reflecting the high concentration of research output in a small number of core journals. Meanwhile, numerous peripheral journals published only sporadic studies, highlighting the centralization of research publication platforms and the academic leadership role of core journals. Therefore, focusing on these core journals significantly enhanced the efficiency and quality of literature retrieval and relevant research information search. This approach helped researchers quickly locate key publications, ensuring a comprehensive and in-depth research foundation.

Figure 5B presents the cumulative publication volume trends of core journals over time. The overall data indicate that 2019–2020 was a critical inflection point for publications in this field: before 2019, cumulative publications of most core journals increased mildly and steadily; after 2019, *Journal of Pediatric Endocrinology & Metabolism* and *Frontiers in Endocrinology* saw a sharp rise in publications, while *Nutrients* maintained slow and stable growth. Among them, *Journal of Pediatric Endocrinology & Metabolism* maintained rapid growth and ranked first in total cumulative publications by 2025. Core journals display divergent developmental trajectories. As a well-established authoritative journal, *International Journal of Obesity* kept the leading cumulative output before 2013 and then plateaued at a high level with limited growth. Pediatric endocrinology specialty journals represented by *Hormone Research in Paediatrics* grew steadily, while *Journal of Pediatric Endocrinology & Metabolism*, *BMC Pediatrics*, *Pediatric Obesity and Pediatric Diabetes* experienced remarkably accelerated growth after 2019.

### 3.4 Keyword trends

**Figure 6:**
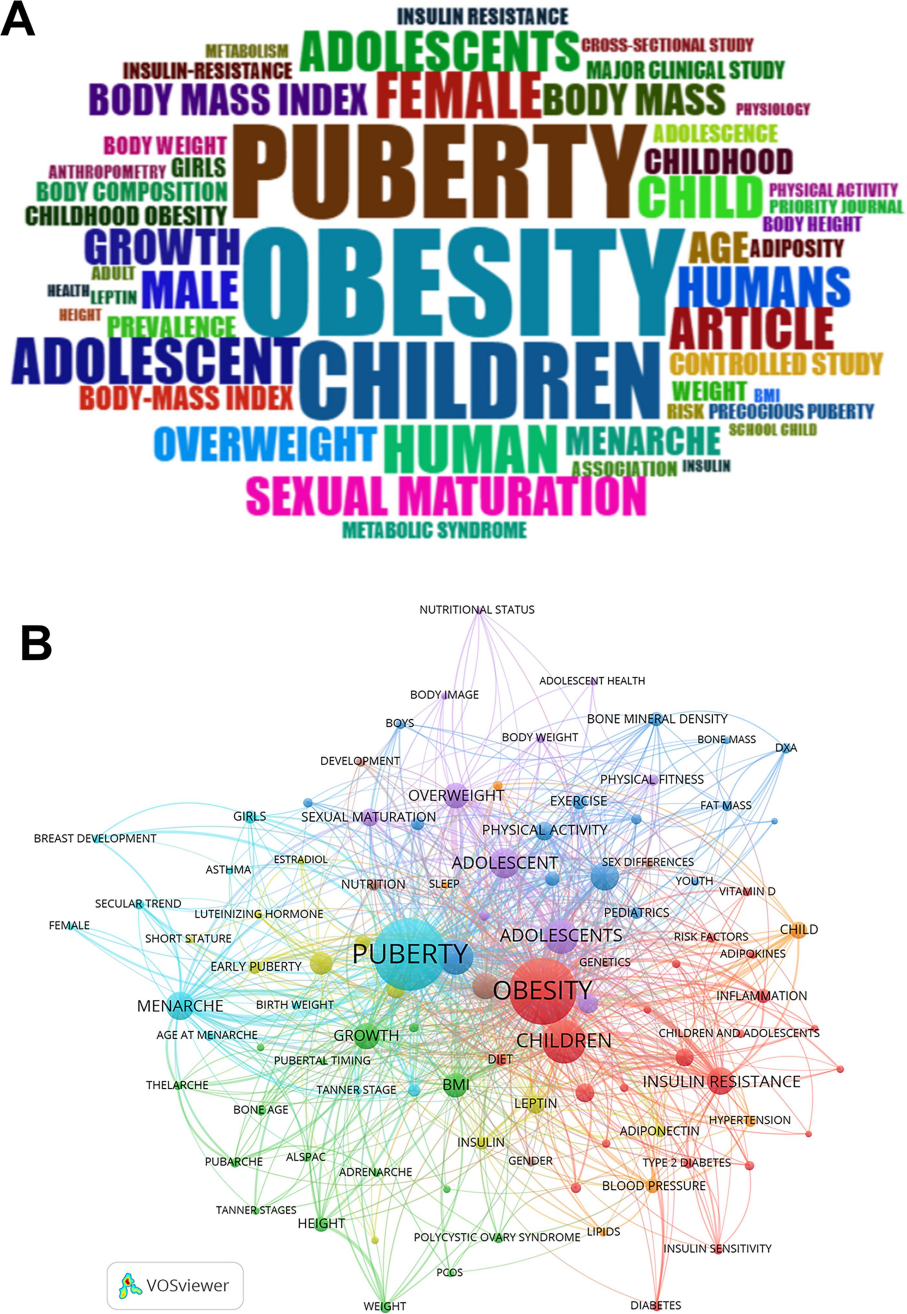
Keyword trends. **(A)** Keyword word cloud: The size of a word indicates its occurrence frequency; the larger the word, the higher the frequency. **(B)** Keyword co-occurrence network: Node size represents keyword frequency, while line thickness reflects co-occurrence strength between keywords.

Keywords served as concise summaries of core arguments, with their frequency and distribution patterns accurately reflecting the overall characteristics, hotspot correlations, and future development directions of a research field. As shown in Figure 6A, Word cloud analysis based on merged keyword frequencies identified two dominant high-frequency terms: “obesity” (1,179 occurrences) and “puberty” (1,031). Demographic keywords including “children” (832), “adolescent” (512) and “female” (513) indicated that previous studies mainly enrolled children and adolescent females as research subjects. High-frequency indicators such as “overweight” (381), multiple expressions of BMI, “sexual maturation” (435) and “menarche” (319) showed that scholars mostly adopted body composition and pubertal developmental indicators for detection. Keywords of “controlled study” (243) and “prevalence” (234) reflected that clinical and epidemiological designs served as the primary research approaches in this field. Overall, existing studies centered on exploring the association between childhood obesity and pubertal development. Researchers relied on clinical epidemiological methods to conduct population investigations and analyzed the effects of obesity on the onset and progression of puberty via anthropometric and sexual developmental measurements.

Co-occurrence networks reveal thematic correlations, research hotspots and collaborative clusters across the analyzed literature. We used VOSviewer to generate a co-occurrence network (Figure 6B), comprising 100 nodes and 1,586 links, with the aim of clarifying the knowledge architecture within this field. “Puberty” served as the dominant node of the entire network, exhibited the highest total association strength and centrality values, and acted as the core hub coordinating research themes across the entire field. Multiple clusters formed around this central node. The largest of these clusters centred on “obesity”, “children” and “BMI”, with dense internal connections, highlighting the field's core research direction: the association between pubertal development and body weight status in paediatric populations. The high-frequency co-occurrence of “insulin resistance”, “leptin” and “metabolic syndrome” within this cluster indicated that a significant body of research approached the subject from metabolic and endocrine pathways, analyzing the intrinsic mechanisms by which body fat levels regulated the timing of puberty onset. Another major cluster centred on the keywords “menarche”, “puberty” and “body composition”, corresponding to a research branch specifically focused on female pubertal development, with a particular emphasis on the relationship between menarche and physical growth, as well as body fat distribution. High-weight nodes such as “precocious menarche” and “central precocious puberty” represented a major clinical research direction, centering on investigations into the prevalence, aetiology and clinical interventions for PP. The network structure revealed that “obesity” and “puberty” were the two core research subjects in this field, with “adolescents” serving as a key bridging node, connecting research on young children with studies on long-term metabolic outcomes. Peripheral terms such as “genetics”, “diet”, “physical activity” and “polycystic ovary syndrome” broadened the scope of research across multiple dimensions, including nutrition, lifestyle and genetic aetiology.

**Figure 7:**
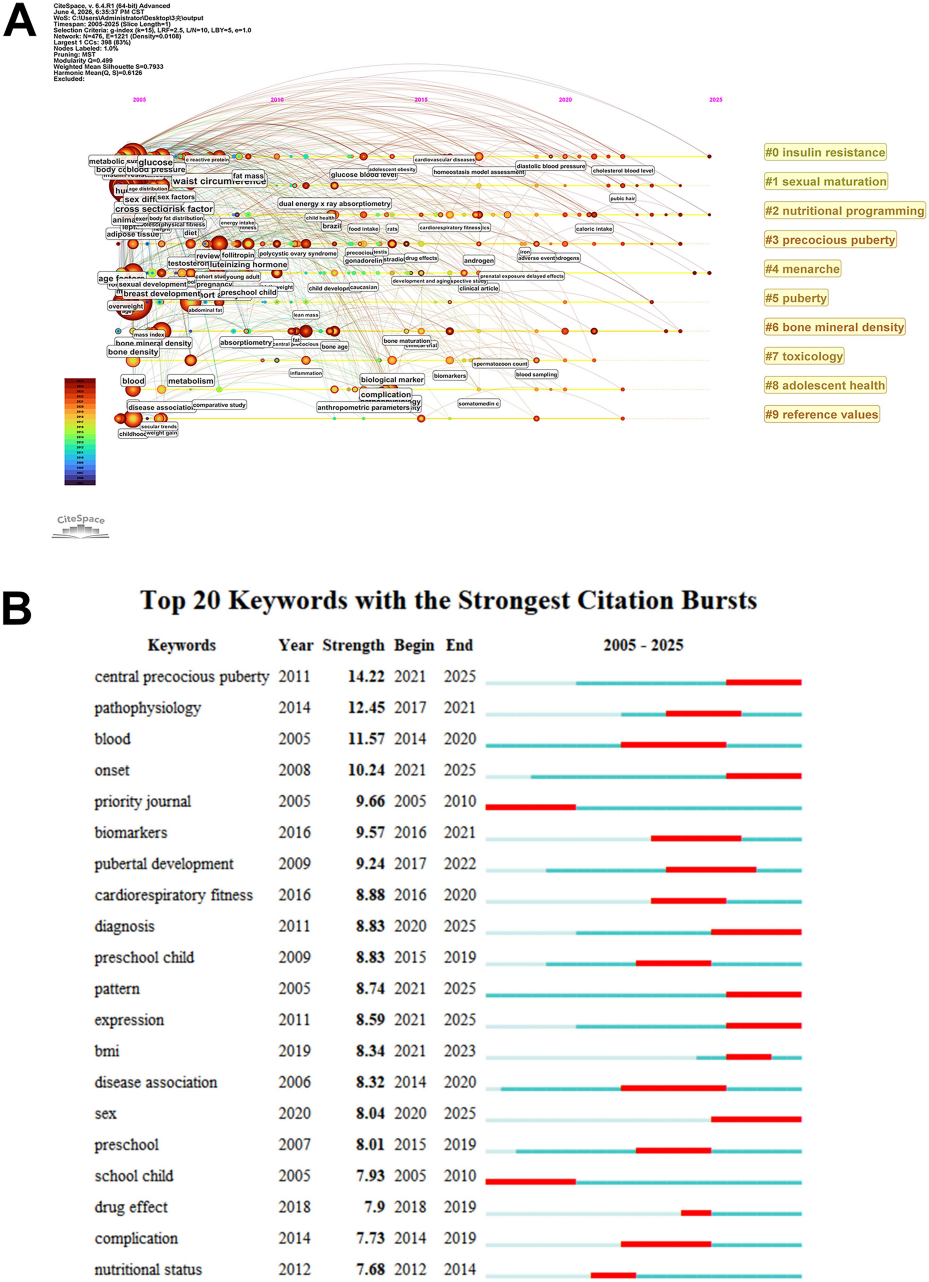
Trends and burst analysis of keywords. **(A)** Timeline view of keywords. The diagram uses circular nodes to denote keywords, where node size corresponds to keyword frequency. Different shades of color indicate the chronological order, and connections between nodes represent the correlations among keywords. **(B)** The top 20 keywords with the strongest citation bursts. The blue lines indicate the time at which the keywords appeared, while the red lines indicate high-frequency citations of the literature during that period. “Strength” is used to quantify the emergence strength of keywords within a specific time frame, reflecting the magnitude of the sudden surge in research interest.

The keywords were visualized and analyzed using CiteSpace, and a timeline view of the keywords was generated (Figure 7A). The timeline displayed high-frequency terms within each cluster across different research stages. The results indicated that keywords focusing on childhood obesity and precocious puberty were classified into ten clusters. Among them, Cluster #0 (insulin resistance) was the earliest core cluster, covering research items such as “insulin secretion” and “metabolic syndrome”, with “body composition” and “fatty acids” serving as basic research themes throughout its development. The typical subsequent cluster #1 (sexual maturation) focused on “pubertal”, “breast development” and “sex differences”, while Cluster #3 (precocious puberty) centred on “central precocious puberty” and clinical intervention with “gonadotropin-releasing hormone analogue”. The remaining clusters, namely #2 (nutritional programming and dietary intervention), #4 (menarche and female developmental characteristics), #5 (overall pubertal development), #6 (bone mineral density and skeletal growth), #7 (toxicology and environmental endocrine disruption), #8 (adolescent comprehensive health), #9 (reference values and physical assessment criteria), covered multiple research fields including dietary nutrition, gonadal development, bone development, environmental toxicology and adolescent health evaluation. As research progressed, new publications and emerging keywords appeared continuously along the timeline, and all ten clusters from #0 to #9 evolved continuously from the early research stage to the present.

Figure 7B presented the top 20 keywords with the strongest citation bursts. Notably, the most popular keywords in recent years were “central precocious puberty”, “onset”, “pattern”, “expression” and “sex”. In contrast, the keyword with the highest burst strength was “central precocious puberty” (14.22), followed by “pathophysiology” (12.45) and “blood” (11.57). The burst strengths of “onset” (10.24) and “biomarkers” (9.57) also exceeded 9.5, reflecting that clinical pathogenesis, puberty initiation characteristics and biological marker exploration were the core focuses of recent research in this field.

### 3.5 LDA-based topic modeling

Based on the LDA modeling analysis, this study identified 15 main potential topics, and all topic labels were determined after manual review of the terminology and representative documents for each topic. As shown in Table 5, the results indicated that the primary themes were Topic 4 “Endocrine & Growth Disorders” (*n* = 226; 8.05%), and Topic 11 “Pediatric Obesity Epidemiology” (*n* = 167; 7.35%), followed by Topic 2 “Adolescent Lipid & Population” (*n* = 193; 7.2%).

**Table 5:** Topics discovered from 2506 articles published between 2005 and 2025.

Topic	Prevalence	Top Terms	Label	Documents
Topic 4	0.0805	abnormal_growth, aromatase_deficiency, endocrine_function, common_endocrine, clinical_findings, estrogen_replacement, gene_mutations, height_fh, growth_model, girls_examined	Endocrine & Growth Disorders	226
Topic 11	0.0735	age-specific, among_school, associated_central, associated_significantly, bmr, bpa_concentrations, girls_reported, girls_stage, gonadarche_boys, hispanic_white	Pediatric Obesity Epidemiology	167
Topic 2	0.072	adolescentes_de, adolescents_city, adolescents_increased, adolescents_presented, cholesterol_p, circumferenceheight, correlated_significantly, cvrf, determine_body, en_adolescentes	Adolescent Lipid & Population	193
Topic 3	0.0715	age_estimation;bmi_rebound; boys_childhood; boys_children; brazilian_population; children_likely; ci_obesity; diurnal_cortisol; glucose_curve; gonad	Child Metabolism & Puberty	201
Topic 6	0.0691	activity_fitness, age_adult, age_obesity, association_among, attained_height, bmi_beta, body_compositions, cpp_risk, cardiac_autonomic, bone_accretion	Child Obesity & CPP Risk	173
Topic 13	0.068	adolescents_obese, ahi, bone_markers, childhoodonset_obesity, combined_hgd, corticosteroid, determine_prevalence, growth_endocrine, higher_ghrelin, hormonal_factors	Child Obesity & CPP-Related Hormones	161
Topic 8	0.0671	adolescents_overweightobesity, analogue_therapy, auxological_biochemical, contributing_factors, dodm, equol, fried, insulinresistance, levels_lh, levels_igfigfbp	Obesity & Pubertal Hormone Links	171
Topic 10	0.0664	accelerated_bone, adiponectin_ghrelin, adult_diseases, antiinflammatory, early_postnatal, early_timing, leptin_mrna, gnrh_neurons, hypercaloric, increase_adiposity	Obesity & Early Puberty Mechanisms	161
Topic 12	0.0662	adolescents_born, adrenarchal, age_aga, artconceived_children, assisted_reproductive, birthweight_childhood, dheas_p, children_earlier, dbp_p, duration_sleep	Adrenarche & Early Life Exposures	168
Topic 9	0.065	adjunctive, adverse_cardiometabolic, associated_overweightobesity, bdnf_levels, bmi_menarche, bodymass, cortisoltestosterone, early_precocious, clonidine, etiological	Obesity-Menarche Timing & Neuroendocrine Mechanisms	162
Topic 14	0.0649	abnormal_anthropometry, across_early, age_spermarche, attainment_puberty, boys_achieved, features_metabolic, glycated_haemoglobin, delayed_sexual, female_breast, developmental_outcomes	Pubertal Development: Sex Differences	146
Topic 5	0.0626	adhd_symptoms, adolescents_dm, adrenal_glands, bone_disorders, daytime_sleepiness, high_dheas, hormone_acth, elevated_alt, lipogenesis, dietary_insulin	Adrenal Function & Metabolic Abnormalities	152
Topic 1	0.0601	acylated_ghrelin, adma, adolescence_associated, afab, bad_eating, environmental_chemicals, cardiometabolic_inflammatory, cardiometabolic_parameters, biomarkers_associated, ethnicity_puberty	Environmental Chemicals & Metabolic Inflammation	165
Topic 7	0.0597	age_entry, associations_parental, bmi_parental, body_growth, british_cohort, dietinduced_obesity, gestation_lactation, girls_followed, greater_prepubertal, growth_reference	Parental Factors & Child Growth Trajectories	143
Topic 15	0.0534	antibiotics, asthma_risk, circulating_leptin, downstream, microbiota_composition, lh_pulse, lh_pulses, nutrition_adolescence, hyperglycaemic, europe_nutrition	Gut Microbiota & Pubertal Hormones	117

**Figure 8:**
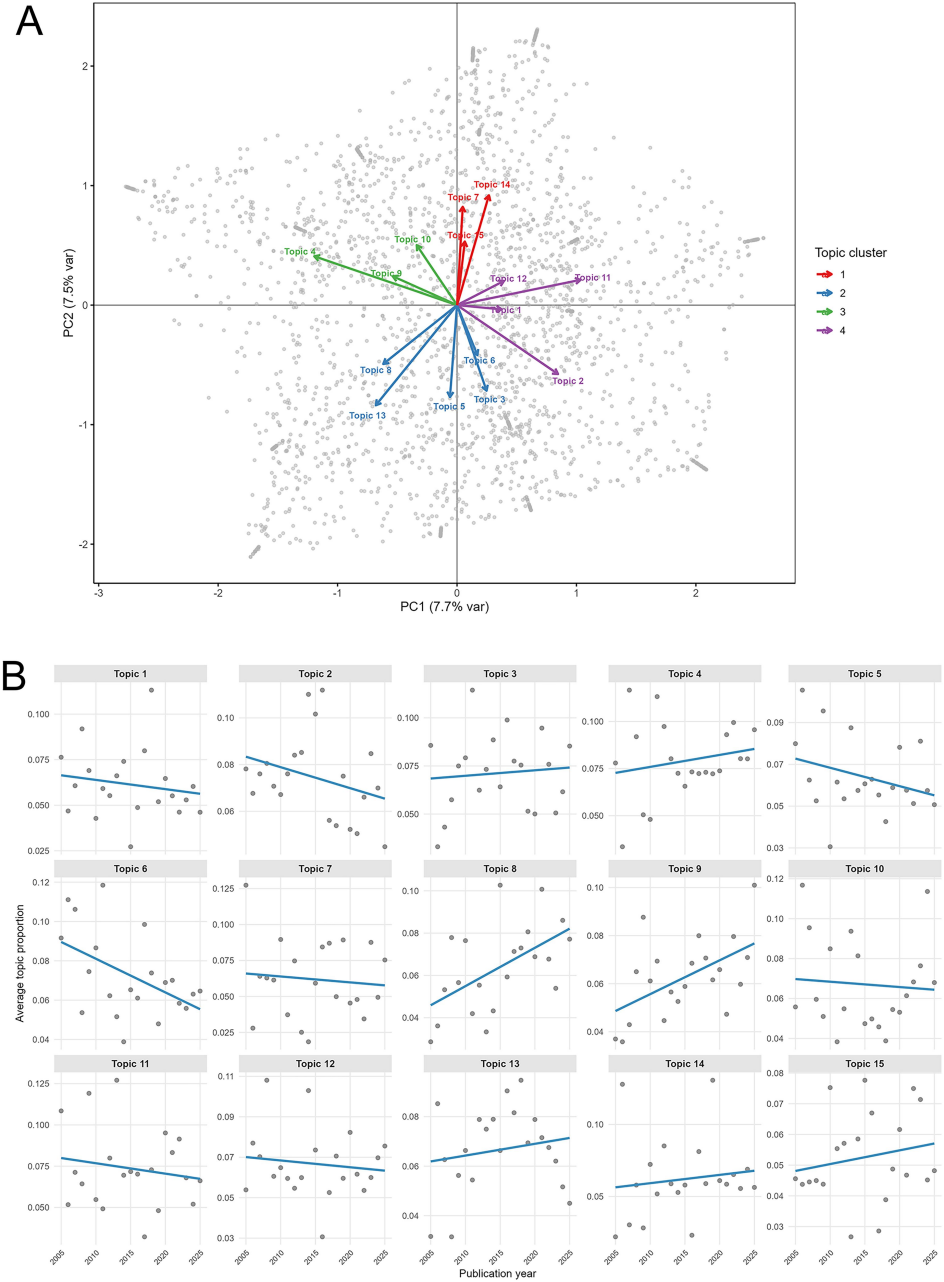
LDA-based topic modeling. **(A)** Two-dimensional visualization of document-topic distribution: Principal component analysis (PCA) based on the topic posterior matrix (*θ*). PC1 and PC2 explain 7.7% and 7.5% of the variance, respectively. Gray dots represent individual documents; colored arrows indicate topic vectors (arrow length represents the contribution to the principal components, and direction represents the strength of association with the axes). All topics are divided into 4 clusters (Clusters 1–4, colors as shown in the legend), which clearly present the intrinsic associations between topics and the distribution structure of documents and topics. **(B)** Temporal trends of latent topics in the field of childhood obesity and precocious puberty (2005–2025): The annual average posterior proportions of 15 topics (Topic 1–Topic 15) are displayed, including annual observations (dots) and fitted linear trends. The direction and magnitude of the slope intuitively reflect the dynamic changes in the research attention of each topic, showing either growth or decline over time. LDA-latent dirichlet allocation.

The document-topic distribution matrix *θ* describes the probability distribution of each paper belonging to different extracted topics. It quantifies the topic composition of individual articles and supports subsequent classification of research documents. The principal component analysis (PCA) of the document-topic distribution matrix *θ* was visualized using a Husson–Jongmans biplot (Figure 8A). The first two principal components explained approximately 7.7% (PC1) and 7.5% of the total variance, respectively. In the biplot, the gray dots represented individual documents, and the colored arrows denoted the topic vectors: longer arrows indicated stronger contributions to the respective components, and documents located in the direction of an arrow corresponded to higher posterior probabilities for that topic. Four distinct topic clusters were identified: Cluster 1 (red): Covered studies on adrenal endocrine function and pubertal physiological processes, including adrenal hormone regulation, sex differences in pubertal timing, and gut microbiota-mediated hormonal signaling pathways. Cluster 2 (blue): Focused on obesity-related metabolic complications and multi-system damage in children, such as insulin resistance, bone metabolism disorders, and long-term cardiovascular risks. Cluster 3 (green): Included core studies on the association between childhood obesity and pubertal timing, as well as neuroendocrine regulatory mechanisms involving leptin, gonadotropin-releasing hormone, and the hypothalamic-pituitary-gonadal axis. Cluster 4 (purple): Centered on upstream etiological factors, including environmental endocrine-disrupting chemicals, dietary behaviors, early-life exposures, and racial/regional disparities in pubertal development.

To analyze the temporal dynamics of research, we fitted linear regression models for each topic, with publication year as the independent variable and the average annual posterior topic proportion as the dependent variable. Trends were classified based on the slope and statistical significance (threshold: *P* < 0.05). Topics showing a significant positive trend included Topic 8 (Obesity and pubertal hormone links; slope = 0.0018, *P* = 0.0109) and Topic 9 (Obesity–menarche timing and neuroendocrine mechanisms; slope = 0.0014, *P* = 0.0132), indicating a significant upward trend in their research prominence. In contrast, Topic 6 (Obesity-related metabolic outcomes; slope = −0.0017, *P* = 0.0247) showed a significant downward trend. Temporal changes in the remaining topics were not statistically significant, indicating relatively stable research interest (Figure 8B). These trend changes were presented in a volcano plot mapping slope versus −log₁₀P (Supplementary Figure S2).

Collectively, the results obtained from CiteSpace timeline analysis (Figure 7A) and VOSviewer keyword co-occurrence network analysis (Figure 6B) are highly consistent with the outputs of the LDA model in this study. The temporal evolution patterns of research clusters presented in the CiteSpace timeline map are well matched to the annual changing trends in topic popularity identified by LDA. Furthermore, the core knowledge structure, mainstream research paradigms, and major research branches reflected in the VOSviewer network further validate that the LDA model was appropriately applied in this study, and that the classified topics have high reliability and stability.

## 4 Discussion

Based on 2,506 relevant publications globally from 2005 to 2025, this study employs bibliometric and visualization analysis to systematically examine the development trajectory and core characteristics of research linking childhood obesity to PP. By examining dimensions such as research trends, global distribution, and thematic evolution, this study provides a scientific reference for future research in this field. The results indicate that academic output is showing a sustained and accelerating growth trend, reflecting the field's increasing public health significance and research importance.

Over the past two decades, it is evident that research into the association between childhood obesity and PP has gone through various phases, in line with global public health trends and technological advances. The exploratory phase (2005–2009) centered on phenotypic and physiological research. Key terms included “obesity”, “puberty”, “sexual maturation”, “body composition”, “waist circumference”, “fat mass” and “leptin”, mainly exploring correlations between adiposity and puberty as well as the function of adipokines. From 2010 to 2017, the field developed steadily. New keywords such as “insulin resistance”, “bone mineral density”, “menarche”, “hypothalamic-pituitary-gonadal axis” and “reproductive hormones” emerged. Research gradually shifted to the mechanisms linking metabolic disorders to PP. Since 2018, driven by multi-omics technologies, relevant research has grown rapidly. Keywords like “environmental exposure”, “toxicology”, “biological markers” and “adolescent health” have gained popularity, making environmental factors and long-term health impacts the new research frontier.

The distribution of global research capacity indicates that research in this field is highly concentrated in North America, Europe and parts of the Asia-Pacific region, with the United States and China accounting for the highest proportion of publications. However, international collaboration networks exhibit marked regional imbalances, with their core primarily concentrated in Europe and North America. This imbalance stems from disparities in research resources, funding and opportunities for international exchange, which may result in global knowledge output failing to adequately reflect the disease characteristics and risk factors of different populations, geographical regions and environmental contexts (27, 28).

Research interests have evolved from basic studies of hormones and epidemiology towards cutting-edge research driven by multi-omics approaches and focused on network-level mechanisms. Based on a systematic review and analysis of the existing literature on relevant mechanisms, this study suggests the potential mechanistic links underlying childhood obesity and PP. A high-calorie diet and a sedentary lifestyle are the primary drivers of childhood obesity (29); these factors induce gut dysbiosis, manifested by a reduction in the abundance of Bifidobacterium and Akkermansia, as well as decreased production of short-chain fatty acids (SCFAs) (30, 31). Microbial dysbiosis further compromises the integrity of the intestinal barrier, acting in synergy with macrophage infiltration and pro-inflammatory polarisation induced by adipose tissue hypertrophy, thereby triggering a systemic state of low-grade inflammation characterised by elevated levels of TNF-α and IL-6 (32, 33). This chronic inflammatory state may exacerbate leptin resistance and insulin resistance (5, 34), thereby creating a vicious cycle at the metabolic level.

At the neuroendocrine regulatory level, leptin, as a key adipokine, exerts a “permissive” regulatory effect on the release of gonadotropin-releasing hormone (GnRH) by binding to leptin receptors on kisspeptin neurons in the arcuate nucleus of the hypothalamus (14, 35, 36); leptin resistance at the metabolic level described above can directly disrupt the normal feedback regulation of the HPG axis, thereby affecting the regulatory balance of gonadal development. Concurrently, insulin resistance can reduce the liver's capacity to synthesise sex hormone-binding globulin (SHBG), leading to elevated levels of free estrogens and androgens. This, in turn, stimulates the secretion of gonadotropins and accelerates the process of gonadal maturation (37, 38), thereby promoting the onset and progression of PP. It is worth noting that this potential pathway also involves a bidirectional positive feedback loop: initial obesity and associated metabolic disorders promote the onset of PP through the aforementioned mechanisms, while the elevated sex hormone levels resulting from PP, in turn, act upon adipose tissue, promoting adipocyte proliferation and lipid accumulation, and may further exacerbate an existing state of low-grade inflammation (39). The exacerbation of inflammation, in turn, worsens gut dysbiosis and metabolic resistance, thereby stimulating the HPG axis more intensely and accelerating the pubertal process (40), forming a self-reinforcing cycle of “obesity–inflammation–PP–more severe obesity and inflammation”.

Despite the growing body of research, numerous challenges remain in this field that require urgent resolution. These limitations are intertwined and not only hinder the advancement of clinical translation and mechanistic research but also undermine the reliability and practicality of the research evidence. This is particularly evident in several key areas, including study design, research methods and intervention studies. The lack of uniform standards for key study endpoints, particularly the inconsistent definitions of PP subtypes (CPP vs. PPP) and the timing of puberty onset (Tanner staging vs. hormonal markers), has led to significant heterogeneity between studies (41). Furthermore, most in-depth mechanistic evidence originates from animal models. Although high-fat diet-induced obesity animal models have successfully replicated the manifestations of PP (42–44), these models struggle to fully simulate the complex circumstances of human children, which are influenced by genetic background, long-term environmental exposure and psychosocial factors. Although detailed mechanisms involving hypothalamic inflammation or specific lipid-sensing pathways have been elucidated in animal studies (45), their precise role in human children remains to be verified. In addition, intervention studies are predominantly observational or short-term in nature, with a lack of large-scale, long-term randomised controlled trials (RCTs) to evaluate the efficacy of multi-target strategies. Existing lifestyle intervention trials suffer from high dropout rates and inconsistent adherence, while pharmacological intervention studies lack assessments of long-term metabolic and reproductive outcomes. For example, although metformin's role in improving metabolic parameters in obese children may be more pronounced in pre-adolescents (46), data on its long-term efficacy and safety in delaying the onset of puberty remain insufficient (47, 48).

Translating findings from mechanistic research into clinical practice is the ultimate goal of research in the field of childhood obesity and PP, and relevant intervention strategies are gradually being refined as scientific understanding deepens. In the future, clinical intervention strategies urgently need to evolve from traditional, relatively broad-brush management approaches toward precision models that target key pathways. Prevention efforts should be shifted further upstream, emphasising comprehensive measures such as prenatal nutrition, gut microbiota colonisation in early life, and reducing exposure to environmental endocrine disruptors during childhood (27, 42, 49). For the paediatric population, the focus should be on creating a supportive health environment, including reducing the intake of processed foods and sugar-sweetened beverages (43, increasing physical activity, and limiting children's exposure to certain environmental endocrine disruptors through policy and public education (27, 44). In terms of treatment, for children diagnosed with CPP, the clinical approach primarily involves pharmacotherapy to improve adult height (50, 51), while weight management is routinely implemented to control obesity itself. For children with obesity-related PP, the intervention model is no longer limited to simple weight loss or GnRH analogue therapy, but focuses on key nodes in the obesity-PP regulatory network to develop targeted, precise intervention strategies. At the level of the gut microbiome, supplementing with specific probiotics or prebiotics to restore microbial balance and increase short-chain fatty acid production represents a promising and relatively safe approach (30). Directly targeting metabolic inflammation—for example, by alleviating low-grade inflammatory states through dietary adjustments or medication—may help improve leptin and insulin sensitivity (52). Metformin, as an insulin sensitizer, has been shown in some studies to potentially play a positive role in delaying the progression of puberty while improving the metabolic status of obese girls (48). At the neuroendocrine level, although the kisspeptin/GPR54 pathway is a key hub for the direct regulation of gonadotropin-releasing hormone neurons (32, 53), the development of its antagonists remains at the preclinical stage (33); nevertheless, it represents a highly specific therapeutic direction for the future. Concurrently, combined exercise interventions have been shown to improve the adipokine profile in obese girls with PP and to delay the progression of puberty (54), demonstrating the benefits of combining lifestyle interventions with physiological mechanisms. In summary, the ideal clinical strategy for the future will be a personalised, comprehensive approach integrating environmental risk avoidance, lifestyle modifications, gut microbiota regulation, and pharmacological interventions targeting specific molecular targets. This paradigm shift from “symptom management” to “mechanism-based intervention” is crucial for fundamentally improving health outcomes in children with obesity-related PP.

## 5 Limitation

This study also has several limitations that warrant careful consideration. Firstly, the literature analysed was primarily sourced from mainstream English-language databases such as WoSCC, Scopus and PubMed; consequently, it may not fully cover high-quality research published in other languages or in regional journals, thereby introducing bias when depicting the global research landscape and may in particular underestimate the research contributions of non-English-speaking countries. Secondly, bibliometric methods essentially analyse the quantity, interrelationships and content of published scientific literature, reflecting research hotspots and knowledge output within the academic community, rather than directly measuring disease burden or clinical efficacy.

Thirdly, this study adopted a conventional static LDA model to clarify the research evolution of this field over the past two decades. Although multidimensional cross-verification combined with CiteSpace timeline mapping and VOSviewer keyword co-occurrence network effectively improved the credibility of thematic clustering results, the LDA model was established merely based on titles and abstracts without full-text information, which remains a major methodological limitation of this study. On the one hand, the unsupervised nature of the LDA algorithm leads to inevitable subjectivity in determining the optimal topic number and interpreting topic labels. Despite clustering verification through principal component analysis, parameter adjustment still causes minor deviations in topic classification. On the other hand, exclusive reliance on bibliographic information omits substantial detailed content from full texts, and the static modeling framework fails to capture subtle and continuous temporal changes in research themes.

Fourthly, this study systematically summarized the research progress of childhood obesity and precocious puberty mainly from biological, metabolic and clinical perspectives, while insufficient attention was paid to ethical, social and psychological dimensions. Current research in this field lacks in-depth discussions on children's psychological impairment, social discrimination and clinical trial ethical specifications, resulting in obvious research gaps and an incomplete overall understanding of this interdisciplinary field. Future studies can further focus on psychosocial and ethical issues to complement the existing research system.

Fifthly, we adopted two statistical approaches for national publication analysis: corresponding author-based counting and full counting. The former attributes each publication to a single country and avoids duplicate counts from international collaboration, while the latter tends to overestimate the output of countries with frequent cooperation, leading to discrepancies in national rankings between the two methods. For institutional analysis, only the full-counting method was used. All institutions involved in collaborative publications are included in the statistics. This approach inflates the publication volume of closely cooperating institutions and creates biases in institutional rankings, an issue that is particularly evident for institutions participating in numerous joint studies. Future research may adopt fractional counting for cross-verification at both national and institutional levels to improve the objectivity of evaluation results.

Sixthly, all journal impact factors referenced in this study are the 2025 Journal Impact Factor. Using data from a single year may bring potential assessment bias, as the impact factors of many journals experience considerable annual fluctuations affected by citations, research hotspots and publishing strategies. A single-year indicator cannot fully reflect the long-term academic influence of journals, and this limitation should be noted when interpreting relevant journal rankings and characteristics.

Finally, this study focused on exploring the overall structure and development patterns of the field, without conducting an in-depth evaluation of the methodological quality or level of evidence of individual studies. Future research could integrate systematic review methods to conduct a graded assessment of the strength of evidence for core mechanisms.

## 6 Conclusion

This study provides a comprehensive review of the key trends and core areas in global research on childhood obesity and PP from 2005 to 2025. It details the research progress made over the past two decades, identifying the core characteristics of this field: a sustained increase in the volume of publications, an uneven regional distribution, and a shift in research focus from classical physiological mechanisms to multidimensional, cutting-edge areas. The analytical framework constructed in this study also offers relevant researchers a clear roadmap for advancing their work. By systematically reviewing the aforementioned developmental trajectory, this study further highlights the field's increasingly significant role in global child public health and pediatric endocrinology. Furthermore, by integrating the current state of research, it identifies future directions: Clinical interventions must shift from traditional broad-spectrum approaches to precision-targeted models focused on key pathways; prevention strategies must be brought forward to early life and prioritize the avoidance of environmental risks; and research into gut microbiota modulation, metabolic and inflammatory interventions, neuroendocrine targets, and lifestyle adjustments must be integrated to establish a personalised, comprehensive, mechanism-oriented intervention system, thereby providing a practical and feasible development pathway for subsequent research and clinical applications. Notably, the standardized research workflow constructed in this study, including multi-database retrieval, standardized duplicates removing, bibliometric indicator analysis, and validated LDA topic modeling, is highly universal and transferable. This analytical framework can be flexibly applied to other pediatric comorbidity studies, such as research on childhood metabolic syndrome, obesity combined with myopia in children, and the correlation between allergic diseases and growth and development.

## Data Availability

Publicly available datasets were analyzed in this study. This data can be found here: Web of Science Core Collection (WOSCC) (https://www.webofscience.com), Scopus (https://www.scopus.com/), PubMed (https://pubmed.ncbi.nlm.nih.gov/).
