# Supplementary material for: Bibliometric and LDA analysis of the correlation between childhood obesity and precocious puberty (2005–2025)
Source: Front Pediatr. 2026 Jul 27;14:1846658. doi: 10.3389/fped.2026.1846658 (PMC13454113; doi:10.3389/fped.2026.1846658)
Supplement: Supplementary file 1 [file Supplementaryfile1.docx]

**Supplementary Files**

**Table S1. Evaluation of all parameter combinations**

| K | α | β | Perplexity | Coherence |
| --- | --- | --- | --- | --- |
| 25 | 0.01 | 0.01 | 808.78 | -1.0669 |
| 25 | 0.01 | 0.005 | 821.7 | -1.2174 |
| 25 | 0.01 | 0.05 | 864.06 | -0.8626 |
| 25 | 0.05 | 0.01 | 887.06 | -1.2367 |
| 25 | 0.05 | 0.005 | 890.55 | -1.32 |
| 20 | 0.01 | 0.01 | 958.51 | -1.0846 |
| 25 | 0.1 | 0.005 | 966.63 | -1.4041 |
| 25 | 0.1 | 0.01 | 967.84 | -1.28 |
| 25 | 0.01 | 0.1 | 978.7 | -0.7234 |
| 20 | 0.01 | 0.005 | 983.84 | -1.1835 |
| 25 | 0.05 | 0.05 | 988.74 | -1.052 |
| 20 | 0.01 | 0.05 | 1015.66 | -0.8618 |
| 20 | 0.05 | 0.01 | 1042.81 | -1.2471 |
| 20 | 0.05 | 0.005 | 1055.45 | -1.3419 |
| 25 | 0.1 | 0.05 | 1083.11 | -1.1081 |
| 25 | 0.05 | 0.1 | 1108.5 | -0.9461 |
| 20 | 0.01 | 0.1 | 1114.26 | -0.7598 |
| 20 | 0.05 | 0.05 | 1115.05 | -1.0819 |
| 20 | 0.1 | 0.01 | 1118.15 | -1.3481 |
| 20 | 0.1 | 0.005 | 1130.58 | -1.3966 |
| 15 | 0.01 | 0.01 | 1205.47 | -1.1761 |
| 20 | 0.1 | 0.05 | 1205.92 | -1.1518 |
| 15 | 0.01 | 0.005 | 1218.86 | -1.2884 |
| 20 | 0.05 | 0.1 | 1227.88 | -0.9773 |
| 25 | 0.1 | 0.1 | 1237.24 | -1 |
| 15 | 0.01 | 0.05 | 1239.35 | -0.9944 |
| 15 | 0.05 | 0.01 | 1290.8 | -1.2937 |
| 15 | 0.05 | 0.005 | 1296.04 | -1.3711 |
| 15 | 0.05 | 0.05 | 1308.47 | -1.0782 |
| 15 | 0.01 | 0.1 | 1332.78 | -0.9379 |
| 20 | 0.1 | 0.1 | 1340.39 | -1.0014 |
| 15 | 0.1 | 0.01 | 1345.41 | -1.32 |
| 15 | 0.1 | 0.005 | 1386.56 | -1.4315 |
| 15 | 0.1 | 0.05 | 1399.81 | -1.1119 |
| 15 | 0.05 | 0.1 | 1420.2 | -1.0015 |
| 15 | 0.1 | 0.1 | 1508.23 | -1.0005 |
| 10 | 0.01 | 0.01 | 1624.71 | -1.181 |
| 10 | 0.01 | 0.05 | 1638.76 | -1.0483 |
| 10 | 0.01 | 0.005 | 1691.37 | -1.1768 |
| 10 | 0.05 | 0.05 | 1703.59 | -1.2063 |
| 10 | 0.05 | 0.01 | 1704.25 | -1.2961 |
| 10 | 0.01 | 0.1 | 1713.94 | -0.9064 |
| 10 | 0.05 | 0.005 | 1740.89 | -1.3846 |
| 10 | 0.1 | 0.01 | 1760.09 | -1.3255 |
| 10 | 0.1 | 0.05 | 1765.38 | -1.1862 |
| 10 | 0.05 | 0.1 | 1787.2 | -1.1989 |
| 10 | 0.1 | 0.005 | 1829.26 | -1.4216 |
| 10 | 0.1 | 0.1 | 1863.39 | -1.1253 |
| 5 | 0.01 | 0.05 | 2639.52 | -1.0412 |
| 5 | 0.1 | 0.05 | 2677.93 | -1.2144 |
| 5 | 0.05 | 0.05 | 2679.66 | -1.1812 |
| 5 | 0.01 | 0.01 | 2695.4 | -1.2502 |
| 5 | 0.01 | 0.1 | 2704.07 | -1.0831 |
| 5 | 0.05 | 0.1 | 2709.12 | -1.1274 |
| 5 | 0.05 | 0.01 | 2730.43 | -1.2559 |
| 5 | 0.01 | 0.005 | 2741.25 | -1.2029 |
| 5 | 0.1 | 0.1 | 2746.83 | -1.1412 |
| 5 | 0.05 | 0.005 | 2797.67 | -1.2924 |
| 5 | 0.1 | 0.01 | 2851.7 | -1.2499 |
| 5 | 0.1 | 0.005 | 2909.92 | -1.3382 |

**Figure S1.** Determination of the optimal topic number K for the LDA model. (A) Perplexity evaluation; (B) Topic coherence evaluation as a function of K. Different colors denote seven combinations of hyperparameters α and δ.

**Figure S2.** Volcano-style plot of topic trend significance. Each point represents one latent topic; the x-axis is the slope of the topic’s annual mean posterior probability over time (from linear regression), with positive values indicating increasing prominence and negative values indicating decline. The y-axis is –log₁₀(P-value) for the null hypothesis that the slope equals zero (higher values denote stronger statistical evidence of change).
